# Single-Cell Transcriptomic Analysis of the Potential Mechanisms of Follicular Development in *Stra8*-Deficient Mice

**DOI:** 10.3390/ijms26083734

**Published:** 2025-04-15

**Authors:** Han Wang, Qingchun Liu, Shunfeng Cheng, Lan Li, Wei Shen, Wei Ge

**Affiliations:** College of Life Sciences, Key Laboratory of Animal Reproduction and Biotechnology in Universities of Shandong, Qingdao Agricultural University, Qingdao 266109, China; wanghan202206@163.com (H.W.); 15706409117@163.com (Q.L.); sfcheng@qau.edu.cn (S.C.); lli@qau.edu.cn (L.L.)

**Keywords:** *Stra8*, single-cell RNA sequencing, oogenesis

## Abstract

Follicle development is a critical process in mammalian reproduction, with significant implications for ovarian reserve and fertility. *Stra8* is a known key factor regulating the initiation of meiosis; however, oocyte-like cells still appear in *Stra8*-deficient mice. Nevertheless, the underlying mechanism remains unclear and requires further investigation. Therefore, we used single-cell RNA sequencing to construct a comprehensive transcriptional atlas of ovarian cells from both wild-type and *Stra8*-deficient mice at embryonic stages E14.5 and E16.5. With stringent quality control, we obtained a total of 14,755 single cells of six major cell types. A further fine-scale analysis of the germ cell clusters revealed notable heterogeneity between wild-type and *Stra8*-deficient mice. Compared to the wild-type mice, the deficiency in *Stra8* led to the downregulation of meiosis-related genes (e.g., *Pigp, Tex12*, and *Sycp3*), and the upregulation of apoptosis-related genes (e.g., *Fos*, *Jun*, and *Actb*), thereby hindering the meiotic process. Notably, we observed that, following *Stra8* deficiency, the expression levels of *Sub1* and *Stk31* remained elevated at this stage. Furthermore, an RNA interference analysis confirmed the potential role of these genes as regulatory factors in the formation of primordial follicle-like cells. Additionally, *Stra8* deficiency disrupted the signaling between germ cells and pregranulosa cells that is mediated by Mdk–Sdc1, leading to the abnormal expression of the PI3K/AKT signaling pathway. Together, these results shed light on the molecular processes governing germ cell differentiation and folliculogenesis, emphasizing the complex role of *Stra8* in ovarian function.

## 1. Introduction

In mammals, follicular development refers to the process by which ovarian follicles, the basic units of oocyte development [1,2], grow from the primary stage to maturity [3]. During mammalian follicular development, when oocytes enter meiosis and arrest after the first meiotic division, the ovarian pregranulosa cells migrate to surround each oocyte to form primordial follicles [4,5,6]. In mice, primordial follicles form over a period of a week from E17.5 to postnatal day 3–5, and these follicles, in turn, form the ovarian reserve [7,8]. Clinically, the excessive loss of follicles can lead to a reduced ovarian reserve [9] and premature ovarian failure [10,11,12]. However, the mechanisms regulating follicular development remain unclear.

*Stra8* is commonly regarded as a gatekeeper for the transition from mitosis to meiosis [13]. In XX primordial germ cells, *Stra8* expression begins at E12.5 and continues until E16.5, lasting a total of four days [14], which coincides with the timing of meiosis. In contrast, *Stra8* is not expressed in XY primordial germ cells [15,16]. Many researchers have confirmed that the expression of *Stra8* determines the onset of meiosis [17], and that the timing of meiosis affects the differentiation of germ cells towards X or Y [18]. Recent studies have found that *Stra8* initiates meiosis by cooperating with various genes and transcription factors, including retinoic acid receptor (RAR) [19,20,21], retinoblastoma (RB) [22], and MEIOSIN (a meiosis initiator) [23]. In addition, oocytes lacking *Stra8* are unable to enter meiosis normally, and, thus, most of these oocytes exhibit mitotic characteristics during development [22,23]. In particular, in *Stra8*-deficient mice, a significant number of oocytes are lost in the fetal ovaries, primarily due to apoptosis [24]. In DNA meiotic recombinase 1-deficient (*Dmc1*-deficient) mice [25], severe meiotic recombination defects are observed, and no follicles were present in the ovaries of postnatal day 21 mice. Similarly, in meiosis-specific double-strand break-deficient *(Spo11*-deficient) mice [26,27], germ cells fail to undergo homologous chromosome pairing and, again, no follicles were found in the ovaries of neonatal mice. Interestingly, despite the inability to proceed through meiosis, *Stra8*-deficient mice still produced oocyte-like cells [28]. These oocyte-like cells are capable of developing a zona pellucida, producing polar bodies, and apparently progressing through in vitro fertilization. While previous studies have focused on the phenotype of oocyte-like cells in *Stra8*-deficient mice, the underlying mechanisms driving this phenomenon remain unresolved.

Single-cell RNA sequencing (scRNA-seq) technology addresses issues of cellular heterogeneity [29,30] that traditional RNA-seq could not resolve, and has become a powerful tool for studying ovarian follicle development in mice [31]. In this study, we successfully constructed a single-cell transcriptome of the ovaries from *Stra8*-deficient mice using scRNA-seq. Our analysis identified six distinct cell types. Notably, we characterized the gene expression profile of germ cells following *Stra8* deficiency. Specifically, we focused on the key transcription factors involved in the process of folliculogenesis. Additionally, we found that *Stra8* deficiency disrupted the Mdk–Sdc1-mediated signaling between germ cells and pregranulosa cells, resulting in the abnormal expression of the PI3K/AKT signaling pathway. These findings provide novel insights into the role of *Stra8* in germ cell development.

## 2. Results

### 2.1. Construction of Single-Cell Transcriptional Atlases of Ovaries in WT and Stra8-Deficient Mice

**Figure 1:**
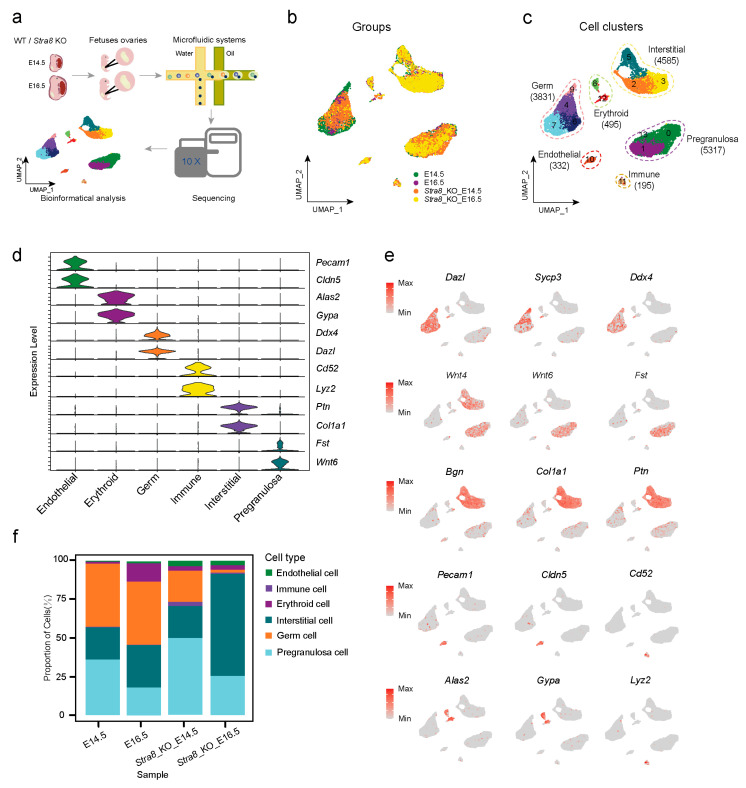
Construction of single-cell transcriptional atlases of ovaries in WT and *Stra8*-deficient mice. (**a**) Schematic diagram of genital ridge collection in WT and *Stra8*-deficient mice at different time points and scRNA-seq analysis. (**b**) Distribution of ovarian cells in E14.5, E16.5, *Stra8-*deficient E14.5 and *Stra8-*deficient E16.5 groups. (**c**) Distribution of ovarian cells in the UMAP plot. The numbers and colors in the figure represent different cell clusters identified by Seurat. (**d**) Violin diagram of signature genes in different cell types. (**e**) Distribution of signature genes across different cell types in the UMAP plot. (**f**) The proportion of different cell types in the four groups.

*Stra8* expression begins at E12.5 (lasting for a total of 4 days), so we selected wild-type (WT) and *Stra8*-deficient mice for ovary collection at two time points: E14.5 and E16.5. The collected ovaries were processed to generate single-cell suspensions for single-cell transcriptome sequencing (Figure 1a). We first performed dimensionality reduction and quality control on four sets of single-cell data, yielding a total of 14,755 cells and 23,223 genes (including 5202 cells from E14.5 WT, 1667 cells from E16.5 WT, 4774 cells from E14.5 *Stra8*-deficient, and 3112 cells from E16.5 *Stra8*-deficient; Figure S1a,b and Table S1). To further characterize the cells, we combined the four quality-controlled datasets and identified a total of 14 clusters. Data spanning the four time points were visualized using Uniform Manifold Approximation and Projection (UMAP) (Figure 1b). We then selected marker genes for different cell types to define their identities, obtaining a total of six cell types: germ cells (3831 cells), interstitial cells (4585 cells), erythroid cells (495 cells), pregranulosa cells (5317 cells), endothelial cells (332 cells), and immune cells (195 cells) (Figure 1c).

To verify the accuracy of cell type annotation, we analyzed and visualized the expression of signature genes for the six cell types. *Dazl* and *Ddx4* were selected as marker genes for germ cells [32,33] (Figure 1d). We then examined the expression of *Dazl* and *Ddx4* across the six cell types (Figure 1d) and found that these genes were highly expressed in clusters 4, 6, 7, and 9 (Figure 1e). Regarding *Fst*, *Wnt4,* and *Wnt6* [34,35], we identified three clusters with a high expression of these genes, which we designated as pregranulosa cells (Figure 1d,e). Using the same approach, we identified the following four additional cell types: endothelial cells (*Pecam1*, *Cldn5*) [36,37], immune cells (*Cd52*, *Lyz2*) [38,39,40], interstitial cells (*Ptn*, *Col1a1*) [41], and erythroid cells (*Alas2*, *Gypa*) [42,43,44] (Figure 1d,e). Following cell type identification, a heatmap was generated to display the highly expressed genes associated with each cell population. Additionally, a gene ontology (GO) enrichment analysis was conducted on differentially expressed genes to elucidate the distinct biological roles of each cell type (Figure S1c). We found that germ cells mainly exhibited enrichment in biological functions related to germ cell development, homologous chromosome segregation, and meiosis. Pregranulosa cells were primarily enriched in the GO terms of “reproductive system development” and “positive regulation of cellular component biogenesis”. Endothelial cells predominantly upregulated specific genes involved in vascular development, which is also consistent with the previous findings [30] (Figure S1c). Based on the above analyses, we determined the accuracy of the single-cell profile and clarified the cellular heterogeneity in ovarian somatic cells. To further investigate the effect of *Stra8* on different cell types, we also analyzed the proportion of cells in each cell type. We observed that *Stra8* deficiency led to a substantial reduction in the proportion of germ cells, while the proportion of interstitial cells significantly increased at E16.5 (Figure 1f). Together, our analysis provides the first single-cell resolution characterization of the effects of *Stra8* deficiency on ovarian somatic cells and the dynamic changes in different cell types over time.

### 2.2. Analysis of Differentially Expressed Genes (DEGs) Among Germ Cell Subsets

**Figure 2:**
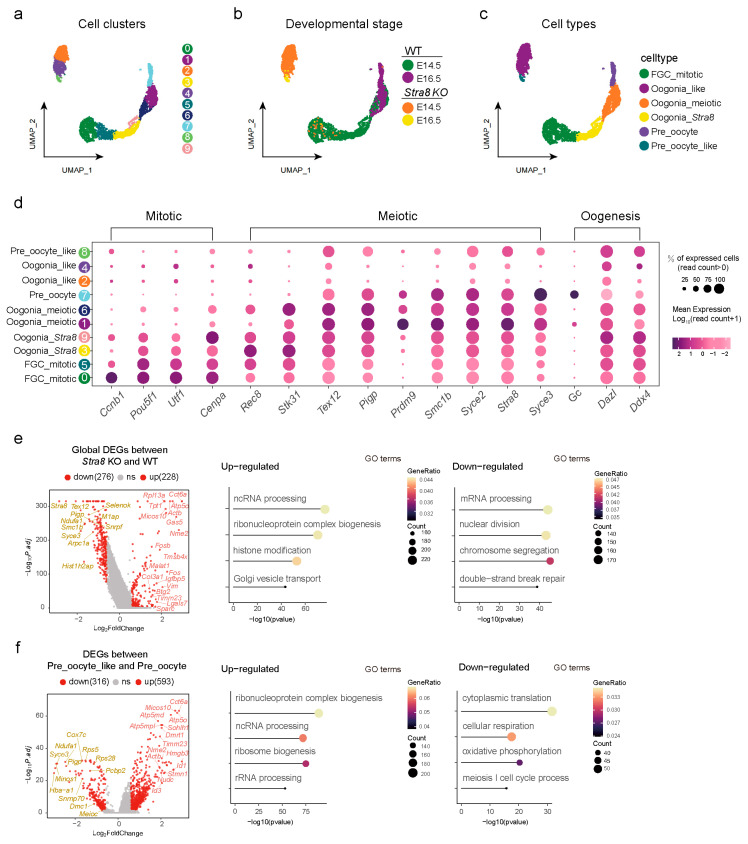
Analysis of differentially expressed genes (DEGs) among germ cell subsets. (**a**) Distribution of germ cell subpopulations in the UMAP plot. The numbers in the figure represent different cell subclusters identified by Seurat. (**b**) UMAP diagram at different developmental stages (E14.5, E16.5, *Stra8-*deficient E14.5, and *Stra8-*deficient E16.5). (**c**) Annotations on germ cell subpopulations (FGC_mitotic, Oogonia_*Stra8*, Oogonia_meiotic, Pre_oocyte, Oogonia_like, and Pre_oocyte_like). (**d**) Dot plot showing germ cell cluster-specifically expressed genes. (**e**) Left panel: volcano plot illustrating global DEGs between *Stra8-*deficient and WT groups (left); right panel: corresponding GO enrichment results of up-regulated and down-regulated genes (right). (**f**) Left panel: volcano plot illustrating DEGs between Pre_oocyte_like and Pre_oocyte cell clusters (left); right panel: corresponding GO enrichment results of up-regulated and down-regulated genes (right).

To gain deeper insights into the role of *Stra8* in germ cells, we conducted a sub-clustering analysis on the three germ cell clusters and generated a UMAP plot (Figure 2a). Following sub-clustering, we identified 10 distinct clusters in total (Figure 2a). Through an analysis of the germ cell gene expression atlas at various time points, we observed dynamic changes in their developmental trajectories over time (Figure 2b). A UMAP analysis revealed distinct clustering between germ cells of *Stra8*-deficient and WT groups, indicating significant differences in the gene expression profiles.

Initially, we visualized the expression patterns of key genes in WT germ cells and observed that *Ccnb1* and *Pou5f1* were predominantly expressed in clusters 0 and 5. *Cenpa* and *Stra8* were predominantly expressed in clusters 3 and 9, while *Tex12* and *Syce2* were mainly enriched in clusters 1 and 6. Lastly, *Pigp* and *Syce3* showed a predominant expression in cluster 7 [45,46] (Figure S2a). Therefore, we annotated clusters 0 and 5 as mitotic fetal germ cells (FGC_mitotic), clusters 3 and 9 as pre-meiotic germ cells (Oogonia_*Stra8*), clusters 1 and 6 as meiotic germ cells (Oogonia_meiotic), and cluster 7 as early oocytes (Pre_oocyte) (Figure 2c). Similarly, we examined the representative patterns of key marker genes in the *Stra8*-deficient group. *Rec8* and *Stk31* were primarily expressed in clusters 2 and 4, leading to their annotation as meiotic-like germ cells (Oogonia_like). Meanwhile, *Smc1b* and *Sycp3* were predominantly expressed in cluster 8. To distinguish them from the WT group’s Pre_oocyte, we annotated them as early oocyte-like cells (Pre_oocyte_like) (Figure 2c and Figure S2a). To verify the accuracy of cell annotations, we generated a dot plot using germ cells from different clusters with highly expressed genes (Figure 2d). The dot plot results were consistent with the previous findings, further confirming the accuracy of our annotations. Through these analyses, we achieved the precise subpopulation classification of germ cells and accurately identified their respective cellular identities.

To further investigate the impact of *Stra8* deficiency on germ cell development, we conducted a stage-specific analysis of differentially expressed genes across various developmental phases (Figure 2e,f). Initially, we examined the overall differences in gene expression profiles between the *Stra8*-deficient and WT groups. We observed that the expressions of *Stra8* (a key “gatekeeper” of meiosis), *Tex12* (a component of the synaptonemal complex), and *Smc1b* (encoding a DNA recombination protein) were significantly downregulated in the *Stra8*-deficient group. In contrast, the expression levels of Fos family genes, including *Fos*/*Fosb* (encoding leucine zipper proteins) and *Actb* (encoding actin), were consistently upregulated in the *Stra8*-deficient group (Figure 2e, left panel). Following this, we utilized a GO enrichment analysis to assess the functional attributes of genes exhibiting both increased and decreased expression levels. We found that the downregulated genes were primarily associated with biological processes such as “nuclear division”, “chromosome segregation”, and “double-strand break repair”. These analyses demonstrated that *Stra8* deficiency leads to a marked downregulation of meiosis-related genes, ultimately impairing meiotic progression, which is consistent with previous studies [47] (Figure 2e, right panel). We then conducted a differential gene analysis between Pre_oocyte and Pre_oocyte_like. We found that *Syce3* (a component of the synaptonemal complex), *Dmc1* (essential for homologous recombination during meiosis), and *Meioc* (promoting the transition from mitosis to meiosis) were consistently downregulated in the *Stra8*-deficient group. In contrast, *Dmrt1* (which inhibits meiosis in male spermatogonia by suppressing retinoic acid and *Stra8* expression), *Sohlh1* (involved in spermatogenesis, oogenesis, and folliculogenesis), and *Id3* (participating in cell growth, differentiation, and apoptosis) were persistently upregulated in the *Stra8*-deficient group (Figure 2f, left panel). Following this, a GO enrichment analysis revealed that the downregulated genes were primarily associated with the GO terms of “oxidative phosphorylation” and “meiosis I cell cycle process” (Figure 2f, right panel). These results further confirmed that, following *Stra8* deficiency, germ cells were unable to properly enter meiosis. Additionally, we performed a Kyoto Encyclopedia of Genes and Genomes (KEGG) enrichment analysis on the differentially expressed genes (Figure S2b). A KEGG analysis in the WT group showed enrichment in pathways such as “DNA replication”, “Oocyte meiosis”, and “Cell cycle”, aligning with the biological processes active at the time of sequencing. In contrast, the *Stra8*-deficient group showed enrichment in pathways such as “Mitophagy”, “Apoptosis”, and “Cellular senescence” (Figure S2b). Together, our analyses here indicated that *Stra8* deficiency not only impaired the initiation of meiosis during meiotic prophase by downregulating meiosis-related genes but also promoted apoptosis and autophagy, especially in germ cells.

### 2.3. Differential Analysis of Germ Cell Fate Trajectories Between WT and Stra8-Deficient Mice

**Figure 3:**
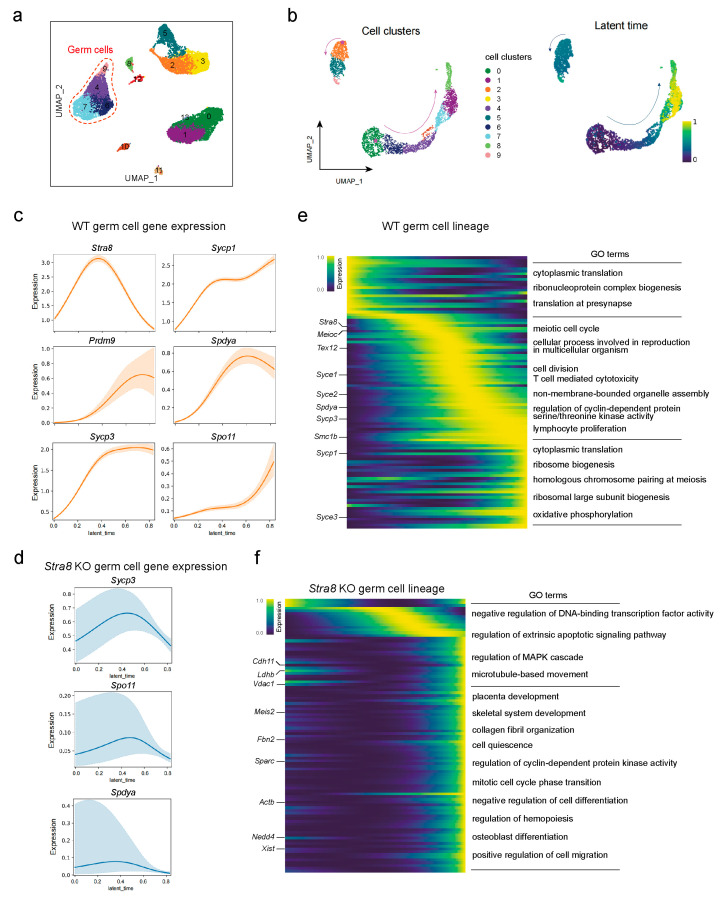
Differential analysis of germ cell fate trajectories between WT and *Stra8-*deficient mice. (**a**) Distribution of three clusters of germ cells in the UMAP plot. The numbers in the figure represent different cell subclusters identified by Seurat. (**b**) Pseudo-temporal trajectory of germ cell development (left) and distribution of germ cells in latent time (right). (**c**) Pseudo-temporal developmental trajectories of representative meiotic genes in the WT group. (**d**) Pseudo-temporal developmental trajectories of representative meiotic genes in *Stra8-*deficient group. (**e**) Expression trend of the top 50 genes in latent time and GO term enrichment in the WT group. (**f**) Expression trend of the top 50 genes in latent time and GO term enrichment in the *Stra8-*deficient group.

Based on the above analyses, we identified the germ cell populations and described the expression patterns of key genes in germ cells (Figure 3a). Next, we used CellRank to reconstruct the differentiation trajectories of germ cells in both WT and *Stra8*-deficient groups, allowing us to infer the transitions between cell states [48]. We observed two distinct developmental patterns in the germ cells of both WT and *Stra8*-deficient groups (Figure 3b). In the WT group, a CellRank analysis identified the FGC_mitotic cluster 0 as the initial state and the Pre_oocyte cluster 8 as the terminal state of cell fate, with arrows indicating the direction of cell differentiation (Figure 3b, left panel). This trajectory aligns with the expected relationship between cell development and differentiation, further validating the accuracy of the cell type annotations (Figure 3b, left panel). Furthermore, the trajectory of the *Stra8*-deficient group started at Oogonia_like cluster 2 and ended at Pre_oocyte_like cluster 9 (Figure 3b, right panel). These results were consistent with our earlier analyses and provide additional evidence for the process of differentiation in germ cells.

Next, we examined the expression changes in key meiosis-related genes along latent time in both the WT and *Stra8*-deficient groups (Figure 3c,d and Figure S3a,b). Our findings indicated a decline in the expression of genes like *Sycp3*, *Sycp1*, *Spdya*, and *Meioc* over latent time in the *Stra8*-deficient group. Subsequently, we analyzed the molecular features and gene dynamics of latent time along germ cell development in both the WT and *Stra8*-deficient groups (Figure 3e,f). In WT group, the early-stage genes were primarily enriched in processes such as “ribonucleoprotein complex biogenesis”. During the intermediate stage, genes were enriched in processes like the “meiotic cell cycle” and “cellular process involved in reproduction in multicellular organism”. At the late stage of differentiation, the genes were predominantly associated with “oxidative phosphorylation” (Figure 3e). While, in the *Stra8*-deficient group, the differentiation trajectory was divided into two stages. At the start point, the genes were enriched in processes including the “regulation of extrinsic apoptotic signaling pathway” and “regulation of MAPK cascade”. At the late stage, the genes were enriched in processes including the “mitotic cell cycle phase transition” and “negative regulation of cell differentiation” (Figure 3f). Overall, our data demonstrated the continuous genetic changes in germ cells between E14.5 and E16.5, providing theoretical support for the *Stra8*-deficiency-mediated meiotic bypass and its associated effects.

### 2.4. Construction of Gene Co-Expression Networks to Identify Key Genes Involved in Follicle Formation

**Figure 4:**
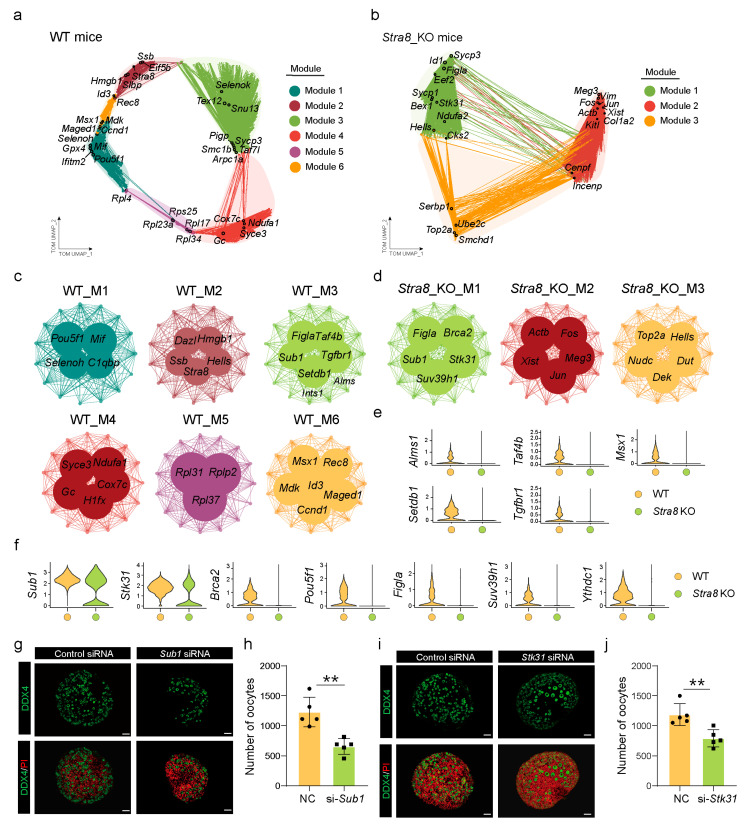
Construction of gene co-expression networks to identify key genes involved in follicle formation. (**a**) UMAP plot of the co-expression network of germ cells from WT mice. (**b**) UMAP plot of the gene co-expression network in germ cells from *Stra8-*deficient mice. (**c**) Gene co-expression network diagram of different modules in the WT group. (**d**) Gene co-expression network diagram of different modules in the *Stra8-*deficient group. (**e**) Violin plots of transcripts that disappear after *Stra8* elimination. (**f**) Violin plots showing genes with decreased expression following *Stra8* deficiency. (**g**) Representative images of ovaries in the control and *Sub1* siRNA groups cultured for six days. Germ cells are stained with DDX4 (green) and nuclei are stained with PI (red). (**h**) The number of oocytes in the control and *Sub1* siRNA groups. (**i**) Representative images of ovaries in the control and *Stk31* siRNA groups cultured for six days. Germ cells are stained with DDX4 (green) and nuclei are stained with PI (red). (**j**) The number of oocytes in the control and *Stk31* siRNA groups. The percentage of each group is presented as the mean ± SD. All experiments were repeated at least three times (** *p* < 0.01).

To elucidate the molecular mechanisms underlying follicle formation in mice with *Stra8* deficiency, we performed an hdWGCNA analysis separately on the WT and *Stra8*-deficient groups to identify co-expression gene modules associated with follicular development in each condition. We found that the WT network consisted of six modules, while the *Stra8*-deficient network consisted of three modules (Figure 4a,b and Figure S4a,b). To identify specific co-expression modules in WT and *Stra8*-deficient groups, we analyzed the expression patterns of the modules in both groups. Additionally, we examined the module eigengenes (MEs) for each module, which provided insights into the role of these modules in follicle development (Figure 4c,d).

Notably, in the WT group, *Pou5f1* and *Selenoh* were highly expressed in Module 1; Module 2 predominantly enriched genes such as *Dazl*, *Stra8*, and *Hells*; while Module 3 enriched genes associated with follicle development, including *Figla*, *Sub1*, and *Taf4b* (Figure 4c). A GO enrichment analysis revealed that Module 1 was associated with the “mitotic cell cycle”, Module 2 was enriched in “DNA replication” and “meiotic cell cycle”, and Module 3 was primarily related to “meiosis I cell cycle process” and “female gamete generation” (Figure S4c). Using the same approach, we analyzed the co-expression modules in the *Stra8*-deficient group. Module 1 exhibited a high expression of follicular development-related genes such as *Figla*, *Sub1*, and *Stk31*. Meanwhile, genes associated with apoptosis, including *Fos*, *Jun*, and *Actb*, were highly expressed in Module 2 (Figure 4d). Next, we performed a GO enrichment analysis for the characteristic genes within these modules. The results revealed that the genes in Module 1 were significantly enriched in “ATP metabolic process” and “DNA damage response”, while genes in Module 2 were enriched in “chromatin organization” and “regulation of RNA splicing” (Figure S4d). These findings indicate that germ cells showed specific biological functions through distinct co-expression networks in the WT and *Stra8*-deficient groups.

From our analysis, we observed that essential genes involved in follicular development, including *Stk31* [49], *Sub1* [50], and *Figla* [51], were expressed in both groups; however, their expression was significantly lower in the *Stra8*-deficient group when compared with the WT group (Figure 4f). Additionally, certain genes crucial for follicle development, including *Taf4b* [52], *Tgfbr1* [53], and *Setdb1* [54], were not expressed in the *Stra8*-deficient group (Figure 4e). Therefore, we hypothesize that *Stra8*-deficient mice have the ability to undergo follicle formation due to the expression of follicle-formation-related genes.

To verify whether these key genes influence follicle development, we performed an RNA interference (RNAi) analysis in vitro. It is widely recognized that primordial follicle formation in mice occurs between E17.5 and postnatal day 3. Based on this, we cultured fetal ovaries from E16.5 fetuses in vitro and transfected them with si-*Sub1* and si-*Stk31*, respectively (Figure 4g–j). The results of a real-time quantitative PCR (RT-qPCR) showed that RNAi effectively reduced the expression level of *Sub1* and *Stk31* in the ovaries (Figure S4e,f). Additionally, we performed immunostaining using a DDX4 antibody to assess the number of primordial follicles in cultured ovaries in vitro. Notably, the number of primordial follicles in the *Sub1*-RNAi and *Stk31*-RNAi groups was significantly decreased when compared to the control group (Figure 4g–j). Specifically, *Sub1*-RNAi led to a decrease in follicle formation and an increase in the proportion of cysts, suggesting that *Sub1*-RNAi impaired cyst breakdown, thereby hindering the formation of primordial follicles (Figure S4g). In contrast, *Stk31*-RNAi reduced the number of primordial follicles but did not affect the follicle-to-cyst ratio (Figure S4h). Finally, we examined the expression of other transcription factors associated with primordial follicle formation using RT-qPCR. As expected, these oocyte-specific transcription factors were notably downregulated in both the *Sub1*-RNAi and *Stk31*-RNAi groups (Figure S4i,j). Together, our findings here indicate that genes associated with follicle formation, including *Sub1* and *Stk31*, play a vital role in the establishment of primordial follicles, which may also explain why *Stra8*-deficient mice are still capable of forming follicles.

### 2.5. Decreased Mdk–Sdc1 Ligand–Receptor Signaling Affected Primordial Follicle Formation in Stra8-Deficient Mice

**Figure 5:**
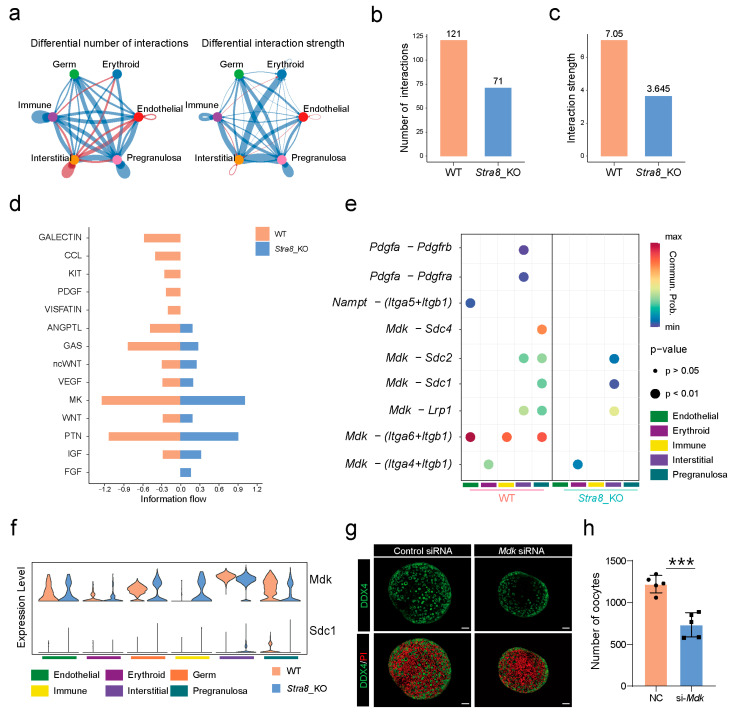
Decreased Mdk–Sdc1 ligand–receptor signaling affects primordial follicle formation in *Stra8*-deficient mice. (**a**) Ovarian cell signaling network. (**b**) The number of interactions between different cell types. (**c**) The interaction strength between different cell types. (**d**) The information flow of WT and *Stra8-*deficient groups. (**e**) The ligand–receptor pairs between germ cells and other somatic cells. (**f**) Expression of ligands and receptors between WT and *Stra8-*deficient groups. (**g**) Representative images of ovaries in the control and *Mdk*–siRNA groups cultured for six days. Germ cells are stained with DDX4 (green) and nuclei are stained with PI (red). (**h**) The number of oocytes in the control group and *Mdk*–siRNA group. The percentage of each group is presented as the mean ± SD. All experiments were repeated at least three times (*** *p* < 0.001).

To investigate the impact of *Stra8* deficiency on ovarian somatic cell communication networks, we used CellChat to construct interaction networks among germ cells, pregranulosa cells, endothelial cells, interstitial cells, erythroid cells, and immune cells. First, we generated a chord diagram to visualize the interactions between different cell types. The diagram revealed stronger interactions between pregranulosa cells and germ cells, as well as between immune cells and endothelial cells, compared to other cell types (Figure 5a). Additionally, we detected a notable decline in the frequency and strength of somatic cell interactions in the ovaries following *Stra8* deficiency (Figure 5b,c). We further compared the information flow between the two datasets (Figure 5d). Interestingly, although the activity of each signaling pathway varied, eight out of fourteen pathways were highly active. Five signaling pathways were active only in the WT group, including KIT [55,56], PDGF [57,58], and VISFATIN [59,60], among others, which are associated with the primordial follicle activation and reproductive lifespan in mice. Only one signaling pathway, FGF [61,62,63], was particularly active in *Stra8*-deficient groups.

Subsequently, we examined alterations in ligand–receptor interactions involving germ cells and the surrounding ovarian somatic cells in both the WT and *Stra8*-deficient groups. This analysis uncovered several important ligand–receptor pairs involved in mediating interactions between germ cells and other somatic cells. We next focused on the interaction between germ cells and pregranulosa cells (Figure 5e). The Midkine (MK) signaling pathway was uniquely identified as the primary communication route between germ cells and pregranulosa cells (Figure 5f). Specifically, after a *Stra8* deficiency, the signaling intensity of the Mdk–Sdc4, Mdk–Sdc2, and Mdk–Sdc1 ligand–receptor interactions was significantly decreased. We also evaluated the expression patterns of *Mdk* and *Sdc1*. A high expression of *Mdk* was observed in germ cells, whereas *Sdc1* was predominantly expressed in pregranulosa cells (Figure 5f). Notably, *Sdc1* expression was decreased in pregranulosa cells of the *Stra8*-deficient group (Figure 5f). These data suggest that Mdk–Sdc1 ligand–receptor interaction between germ cells and pregranulosa cells may play a critical role during primordial follicle formation. 

To further validate the role of the Mdk–Sdc1 ligand–receptor pair in the mouse ovaries, we performed *Mdk*–RNAi during an in vitro ovarian culture. The results of RT-qPCR verified that RNAi effectively reduced the expression levels of both *Mdk* and *Sdc1* in the ovaries (Figure S5a,b). Furthermore, we observed that *Mdk*–RNAi significantly affected primordial follicle formation, leading to a marked reduction in their numbers (Figure 5g,h and Figure S5c). These findings suggested that the Mdk–Sdc1 ligand–receptor pair played a crucial role through the MK signaling pathway in the formation of primordial follicles in mice.

## 3. Discussion

In this study, we performed scRNA-seq on the fetal ovaries of WT and *Stra8*-deficient mice to reveal the underlying mechanism by which *Stra8* deficiency affected meiosis and follicle formation. We successfully constructed a single-cell transcriptional profile of *Stra8*-deficient mice (Figure 1) and explored the role of *Stra8* in germ cells. Our analyses revealed delayed meiotic progression in germ cells from *Stra8*-deficient mice, accompanied by the significant downregulation of key meiotic genes (Figure 2 and Figure 3). These results are consistent with previous studies demonstrating that *Stra8* is essential for meiotic initiation in both male and female germ cells [28]. Subsequently, we investigated the impact of *Stra8* deficiency on follicle formation. These results revealed a significant reduction in the expression of follicle-specific markers, including *Figla* [51], *Stk31* [64], *Taf4b* [52], and *Tgfbr1* [65], in the germ cells of *Stra8*-deficient mice (Figure 4). Notably, we identified Mdk–Sdc1 as a key ligand–receptor pair mediating interactions between germ cells and pregranulosa cells (Figure 5). These findings together offer important clues regarding the molecular mechanisms behind the inability of *Stra8*-deficient mice to undergo normal meiosis and produce follicles.

*Stra8* was first identified in 1995 and researchers subsequently began investigating its function [66]. It was discovered that *Stra8* regulates meiosis by upregulating meiosis-related genes [67]. In 2006, Koubov et al. and Bowles et al. found that the concentration of retinoic acid determines the expression of *Stra8*, which, in turn, regulates the pre-meiotic phase of germ cells [21,47]. In 2013, Gregoriy et al. reported that oocytes of *Stra8*-deficient mice could not undergo the homologous recombination of chromosomes; however, they could form oocyte-like cells [28]. These oocyte-like cells were able to extrude polar bodies for in vitro fertilization, although the euploid egg could only develop to the two-cell stage. This finding was confirmed by Ishiguro et al. and Shimada et al. [22,23]. They also observed that germ cells from *Stra8*-deficient mice underwent apoptosis shortly after birth, and that the ovarian reserves of adult mice were prematurely depleted. In our results, we also noted that *Stra8* deficiency led to a significant downregulation of key meiosis-related genes, including *Rec8* [68], *Tex12* [69], *Sycp3* [70], *Spo11* [71], and *Spdy* [72] (Figure 2 and Figure 3). This downregulation of gene expression likely contributed to the delay in germ cells’ entry into meiosis. Our data also showed that the *Stra8*-deficient group was enriched in apoptosis-related signaling pathways (Figure S2). Therefore, we postulate that the reduced number of primordial follicles in the ovaries of *Stra8*-deficient mice is due to the induction of cell apoptosis.

A number of genes involved in early oogenesis have been identified through existing studies, including *Figla* [51], *Sohlh1* [73], *Sohlh2* [74], *Lhx8* [75], *Nobox* [11], *Taf4b* [52], *Yy1* [76], and *Tbpl2* [77]. These genes were also detected in our data. Unfortunately, the gene regulatory networks that regulate oocyte growth and development remain unclear. In 2021, Nobuhiko et al. demonstrated that pluripotent stem cells could be rapidly transformed into oocyte-like cells by overexpressing eight transcription factors (*Figla*, *Sohlh1*, *Lhx8*, *Nobox*, *Stat3*, *Tbpl2*, *Dynll1*, and *Sub1*) [50]. Despite the fact that these cells, which have characteristics similar to oocytes, do not go through meiotic division, they are capable of being fertilized. Moreover, they can proceed with cell division and reach the 8-cell stage of embryonic development. These discoveries also suggest that cells sharing characteristics with oocytes are capable of being reconstituted in vitro.

Based on these studies, we hypothesize that the presence of oocyte-like cells in *Stra8*-deficient mice is due to the retention of transcription factors essential for early oogenesis (Figure 4). Our results support this hypothesis, as we observed that genes such as *Taf4b*, *Tgfbr1*, and *Msx1* were lost following *Stra8* deficiency. However, genes such as *Figla*, *Sub1*, *Stk31,* and *Sohlh1* remained expressed. Additionally, we experimentally validated this by performing the in vitro knockdown of *Sub1* and *Stk31*, and the results were consistent with our hypothesis. The targeted knockdown of *Sub1* and *Stk31* in vitro resulted in a marked decrease in ovarian germ cell numbers, alongside disruptions in cyst breakdown and follicular assembly. Therefore, we concluded that the expression of genes promoting oogenesis is likely responsible for the continued presence of follicles in *Stra8*-deficient mice.

Midkine (Mdk), a protein encoded by the *Mdk* gene, is a multifunctional heparin-binding growth factor [78]. It was initially identified in mouse embryos, where its expression peaks during the mid-embryonic stage and gradually decreases with development [79]. Subsequent research has shown that *Mdk* promotes the proliferation of primordial germ cells by suppressing *Dazl* expression, which, in turn, leads to the downregulation of meiotic gene expression [80]. Syndecan-1 (Sdc1), a receptor for Mdk [81], is predominantly expressed in epithelial cells [82], and regulates biological functions such as cell migration and signal transduction [83]. Tan et al. demonstrated that the downregulation of *Sdc1* in pregranulosa cells reduces their population size, thereby impairing primordial follicle formation. Subsequent mechanistic studies revealed that this phenomenon is attributable to the Mdk–Sdc1-mediated PI3K/AKT signaling pathway [81]. Interestingly, in our study, *Sdc1* expression was significantly downregulated in pregranulosa cells of *Stra8*-deficient mice (Figure 5). Based on this observation, we propose that the decreased expression of *Sdc1* may affect follicle formation through the PI3K/AKT signaling pathway in *Stra8*-deficient mice.

Our research further elucidates that the expression of meiosis-related genes in germ cells is downregulated, while genes associated with follicle development are still expressed in *Stra8*-deficient mice. Although *Stra8* deficiency skips meiosis, it still maintains certain key processes of follicle development. Therefore, we propose that, although oocytes cannot undergo normal meiosis, other regulatory mechanisms can also promote the formation of follicles and support their development. Further analysis revealed that several follicle development genes, such as *Sub1*, *Stk31*, and *Sohlh1*, might assume compensatory functions in the absence of meiosis in *Stra8*-deficient mice. The upregulation of these genes may support follicle formation and development by regulating the ovarian microenvironment or influencing other signaling pathways. Moreover, our results highlight the critical role of Mdk–Sdc1 in folliculogenesis. This finding provides new insights into the function of *Stra8* and its broader significance in ovarian development in mice.

## 4. Materials and Methods

### 4.1. Ethics Statement

All experimental protocols received approval from the Experimental Animal Management Committee of Qingdao Agricultural University (No. SYXK-20220-021).

### 4.2. In Vitro Isolation of Genital Ridges

Pregnant mice were euthanized via cervical dislocation. Subsequently, all fetuses were detached from the uterus and transferred into normal saline for cleaning. Under a stereomicroscope, the fetuses were placed with the abdomen facing up, the abdominal viscera were stripped, and the genital ridges attached to the back were stripped along with the mesonephros and transferred to a new saline drop. The mesonephros were carefully removed with forceps and a 1 mL syringe needle, to obtain complete genital ridges, and the separated genital ridges were cleaned three times with normal saline.

### 4.3. Mouse Genotyping

All mouse genotypes were determined through polymerase chain reaction (PCR) amplification of DNA that was extracted from tail tissue. DNA was obtained by placing mouse tail tissues in a centrifuge tube and adding 50 μL lysate (tail digestion buffer: proteinase K = 50:1), and then bathing in water at 55 °C for 10 min and boiling water at 100 °C for 5 min. Tail DNA was genotyped by PCR amplification using the following primers: F1 (5′-ATATGCAACTGACATTGAACTCCC-3′), R1 (5′-ATGGACCAAAGCTCAGTGATTC-3′), and F2 (5′-CGCATACCCAGTTATACGCTC-3′). PCR conditions were established as described below: an initial denaturation step at 94 °C for 3 min, followed by 35 cycles, each consisting of denaturation at 94 °C for 30 s, annealing at 60 °C for 35 s, and extension at 72 °C for 35 s. The process concluded with a final extension step at 72 °C for 5 min. The obtained PCR products were then electrophoresed on a 2% agarose gel (WT: 380 bp; Mutant: 536 bp), and genotype was identified from the PCR products isolated.

### 4.4. Preparation of Single-Cell Suspensions

In order to acquire single-cell suspension, the isolated embryonic ovaries were torn into small fragments. These fragments were then incubated in a solution containing 0.25% trypsin (Hyclone, SH30042.02, Beijing, China) and 2 mg/mL collagenase (Sigma-Aldrich, C5138, Shanghai, China) at 37 °C for 6–8 min. Subsequently, the tissues were dissociated using a pipette to produce single-cell pellets. The resulting cell suspension was passed through a 40 μm cell strainer (BD Falcon, 352340, Corning, NY, USA) and then washed twice with phosphate-buffered saline (PBS) that contained 0.04% bovine serum albumin (BSA) (Solarbio, A8020, Beijing, China). Cell viability was determined using 0.4% Trypan Blue staining and cells with viability greater than 80% were projected to downstream analysis. Finally, the cell concentration was verified to be 1000 cells/μL to ensure it met the requirements for cell barcoding.

### 4.5. Single-Cell Library Preparation and Sequencing

Once a qualified single-cell sample was obtained, cells suspended in a solution of 0.04% BSA in PBS were loaded onto a 10× Chromium chip. Single-cell gel beads in emulsion were then generated using Single-Cell 3′ Library and Gel Bead Kit V2 (10× Genomics Inc., Pleasanton, CA, USA). Following the manufacturer’s instructions, single-cell RNA-seq libraries were constructed. Subsequently, pair-end 150 bp sequencing was carried out on an Illumina HiSeq X Ten sequencer (Illumina, San Diego, CA, USA).

### 4.6. Raw Data Processing

Preliminary sequencing data were transformed into FASTQ format using CellRanger v3.0 software (https://www.10xgenomics.com/ (accessed on 17 February 2025)) using the 10× Genomics standard sequencing protocol. The 10× Genomics pre-built mouse genome for mm10-3.0.0 (https://www.10xgenomics.com/support/software/cell-ranger/downloads#reference-downloads (accessed on 17 February 2025)) was used as the reference genome. Then, the FASTQ files were aligned to mouse genome reference sequence mm10-3.0.0 using CellRanger. Subsequently, we applied CellRanger for preliminary data analysis and generated a file that contained features.tsv, barcodes.tsv, and matrix.mtx.gz files. Eventually, the output files (pre-process data) were used for downstream visualization analysis.

### 4.7. Single Sample Analysis and Aggregation

Following CellRanger analysis, the gene expression matrices were analyzed using the Seurat single-cell RNA-seq analysis R package (v4.2.2) [84]. In brief, the Seurat object was initially generated according to two filtering parameters: *min. cells =* 3 and *low.thresholds =* 200. In each sample, the count of unique genes detected in every cell (i.e., *nFeature_RNA*) was adjusted. This adjustment aimed to remove empty droplets, dying cells, and potential doublets or multiplets for subsequent analyses. In this experiment, we further used the *DoubletFinder* function to predict and filter the doublets in the single-cell sequencing data of each group. Furthermore, we conducted an additional filtering step to exclude cells that exhibited an abnormal distribution of detected unique genes. Specifically, cells with an excessively high number of unique genes were regarded as potential doublets or multiplets. Subsequently, the obtained Seurat objects from different groups were integrated using the *Merge* function, and the *Harmony* package (v.1.2.0) was used to remove batch effects. After completing the normalization and scaling procedures, UMAP, a visualization method for cell clustering in high-dimensional transcriptomic data, was employed for dimension reduction. All parameters were set to default unless otherwise specified.

### 4.8. Identification of DEGs and Gene Enrichment Analysis

Differential genes were identified using the *FindAllMarkers* and *FindMarkers* functions in Seurat. The genes of interest were then identified and introduced into Metscape [85] (https://metascape.org/gp/index.html (accessed on 17 February 2025)) for GO and KEGG enrichment analyses.

### 4.9. Cell Differentiation Trajectory Analysis

The CellRank tool is based on RNA velocity and is used to depict the fate trajectories of cell development [48]. From previously described computational methods [86], we first used CellRank’s Kernel to calculate the transition matrix based on pseudotime. From this, we obtained a preliminary understanding of the cellular dynamics in this dataset. Next, we calculated the probabilities of cell differentiation into initial, terminal, and intermediate states using GPCCA (Generalized Perron Cluster Cluster Analysis) [87,88], along with the *cr.tl.initial_states()* and *cr.tl.terminal_states()* functions. Then, the expression trends of the genes of interest were calculated using a general additive model (GAM) and the *cr.pl.gene_trends()* function. Finally, we use CellRank’s built-in algorithm to group genes with similar expression trends together and visualize each group separately. For visualization, we selected the top 50 genes from each group using the *cr.pl.heatmap()* function to create heatmaps.

### 4.10. hdWGCNA Constructed a Gene Co-Expression Network

Weighted Correlation Network Analysis (WGCNA) explores the relationship between gene networks and phenotypes by identifying gene modules in the dataset, and determining the core genes within the network [89,90]. To this end, we here used hdWGCNA to construct a gene co-expression network for our scRNA seq data. Briefly, the *MetacellsByGroups* function was used to construct metacells, which reduced the sparsity of the single-cell expression matrix. Subsequently, the *TestSoftPowers* function was utilized to ascertain the optimal soft-thresholding power for the construction of the gene co-expression network. Then, the *ModuleConnectivity* function was used to calculate MEs values across the entire single-cell dataset, which determined the likelihood of hub genes. Finally, the *ModuleUMAPPlot* function was employed to map multiple gene network modules onto a UMAP plot. hdWGCNA can reduce sparsity in single-cell transcriptomic data while preserving cellular heterogeneity [91]. The genes used to construct hdWGCNA were those expressed in at least 5% of the cells. The *MetacellsByGroups* function was then used to construct metacells, which reduced the sparsity of the single-cell expression matrix. Subsequently, the *TestSoftPowers* function was utilized to ascertain the optimal soft-thresholding power for the construction of the gene co-expression network. In this experimental setup, we identified the optimal soft-thresholding powers, which were 16 for WT and 2 for the *Stra8* deficient group (Figure S4a,b). Then, the *ModuleConnectivity* function was used to calculate MEs values across the entire single-cell dataset, which determined the likelihood of hub genes. Finally, the *ModuleUMAPPlot* function was employed to map multiple gene network modules onto a UMAP plot.

### 4.11. Interactions Between Different Cell Types

Cellular communication has become a new tool for studying interactions between cells [92], exploring the tumor immune microenvironment [93], and identifying therapeutic targets for diseases [93]. In this study, Seurat was used as the research framework, and a CellChat object was created using CellChat (v.1.6.1). The functions *computeCommunProb* and *aggregateNet* were used to investigate the number and strength of interactions between different cell types. Additionally, the *computeCommunProbPathway* function was applied to calculate the communication outcomes of all ligand–receptor interactions for each signaling pathway.

### 4.12. In Vitro Ovarian Culture

Mouse ovarian tissues were harvested and subsequently rinsed three times in physiological saline. The ovaries were placed on 2% agar blocks and transferred to a 6-well plate (NEST Biotechnology, 703001, Wuxi, China). A complete culture medium was added to the wells, ensuring the medium level was flush with the agar block surface. The complete culture medium consisted of MEM Alpha (Gibco, 12571063, Beijing, China), DMEM/F12 (Gibco, C11330500BT, Beijing, China), and sodium pyruvate (Gibco, 11360070, Beijing, China), supplemented with 10% fetal bovine serum (FBS, Gibco, 10099-141, Beijing, China), and 1% penicillin–streptomycin (HyClone, SV30010, Beijing, China). The culture medium was half-changed every other day, and the culture was maintained for six days.

### 4.13. In Vitro siRNA

Fetal mouse ovaries were isolated from E16.5 embryos and washed with physiological saline. Before transfection, transfection reagents and Lipofectamine™ 3000 (Lipo 3000, Thermo Fisher Scientific, 18324010, Waltham, MA, USA) were separately added to the basal medium and incubated for 5 min. The transfection reagents were then thoroughly mixed with non-sense siRNA (Si-NC) or specific siRNA and allowed to stand for 15 min. A 24-well plate (NEST, 702001, Wuxi, China) was prepared with 300 μL of basal medium per well and incubated in a humidified incubator for 15 min. The washed ovaries were transferred into a 24-well plate, and 100 μL of the transfection mixture was added to each well. Transfection was carried out for 6–8 h. After 8 h, the medium was replaced with a complete culture medium. The culture medium was half-changed every other day, and the culture was maintained for six days.

### 4.14. Ovarian RNA Extraction and Real-Time Quantitative PCR

Ovaries from both control and treatment groups were collected, with a minimum of six ovarian samples per group. Following the manufacturer’s protocol, RNA was extracted using a SPARKeasy Tissue/Cell Rapid RNA Extraction Kit (Sparkjade, AC0202-B, Shandong, China) and subsequently reverse-transcribed into cDNA using a SPARKscript II RT Plus Kit (with gDNA Eraser) (Sparkjade, AG0304-B, Shandong, China). Real-time quantitative PCR (RT-qPCR) was performed using 2× SYBR Green qPCR Mix (with ROX) on a CFX96 Touch Real-Time PCR Detection System (Bio-Rad, Hercules, CA, USA). The RT-qPCR data were analyzed using the 2^(−ΔΔCt)^ method, with *Gapdh* serving as the internal control for normalization.

### 4.15. Ovarian Immunofluorescence

The immunofluorescence protocol has been previously described [31]. Ovarian tissues that had undergone a 6 d culture period were rinsed three times in physiological saline. They were then fixed overnight in 4% paraformaldehyde (Solarbio, P1110, Beijing, China). Following rinsing and dehydration, the samples were embedded in paraffin. Subsequently, the ovarian tissues were sectioned at a thickness of 5 μm using a microtome. Paraffin-embedded sections were treated with a blocking solution at 37 °C for a duration of 45 min. Subsequently, these sections were incubated with the primary antibody at 4 °C overnight. The primary antibody was DDX4 (Abcam, ab27591, Shanghai, China), with a dilution ratio of 1:200. Afterwards, the sections were subjected to an incubation process with the secondary antibody at 37 °C for 45 min. The secondary antibody was Alexa Fluor^®^ 488 (Abcam, ab150077, Shanghai, China), with a dilution ratio of 1:200. Nuclei were stained with PI (Beyotime, ST1569, Shanghai, China), and the slides were then mounted with an anti-fade reagent. Additionally, germ cells that were connected in groups of more than two cells were classified as nest germ cells in ovarian sections, while single, unconnected germ cells were categorized as follicle germ cells. The proportion of germ cells present within nests or follicles was then calculated.

### 4.16. Data Analysis

In this study, the experimental results are presented as the mean ± standard deviation (SD), with at least three independent replicates per group. Statistical analysis was performed using GraphPad Prism 8 software, and differences were assessed by *t*-test to determine statistical significance. Significance was determined as follows: *p* < 0.05 was considered significant, *p* < 0.01 was considered highly significant, and “ns” denoted no significance.

## Data Availability

All single-cell RNA-sequencing data have been deposited in the Genome Sequence Archive in the National Genomics Data Center, China National Center for Bioinformation/Beijing Institute of Genomics, and Chinese Academy of Sciences (Accession number: CRA012559) that are publicly accessible at https://ngdc.cncb.ac.cn/gsa (accessed on 17 February 2025).

## Supplementary Materials

The following supporting information can be downloaded at: https://www.mdpi.com/article/10.3390/ijms26083734/s1.
