# Supplementary material for: Single-Cell Transcriptomic Analysis of the Potential Mechanisms of Follicular Development in Stra8-Deficient Mice
Source: Int J Mol Sci. 2025 Apr 15;26(8):3734. doi: 10.3390/ijms26083734 (PMC12027774; doi:10.3390/ijms26083734)
Supplement: Supplementary file 1 [file ijms-26-03734-s001.zip › Captions for Supplementary figures and table.pdf]

Supplementary Figure S1. Single-cell transcriptome sequencing and cell type annotation of ovarian cells.
(a) Violin plots displaying the number of genes (nGene), counts of mRNA (nUMI), and percentages of mitochondrial mRNA (percent.mt) from the four samples. (b) UMAP plot of ovarian cells in four samples. (c) Heatmap illustrating representative cell-cluster marker gene expression and GO enrichment results of cell cluster-specifically expressed genes.
Supplementary Figure S2. Marker gene expression and KEGG enrichment analysis in germ cells.
(a) UMAP plots of marker genes in germ cells at different developmental stages. (b) KEGG enricment of DEGs identified from the WT and *Stra8*-deficient groups.
Supplementary Figure S3. Gene expression trends of representative meiotic genes in the WT and *Stra8*-deficient groups.
(a) Expression trends of representative genes in the WT group. (b) Expression trends of representative genes in the *Stra8*-deficient group.
Supplementary Figure S4. The hdWGCNA analysis and differential gene verification in the WT and *Stra8*-deficient groups.
(a) The hdWGCNA soft threshold in the WT group. (b) The hdWGCNA soft threshold in the *Stra8*-deficient group. (c) GO enrichment of highly expressed genes in six modules from the WT group. (d) GO enrichment of highly expressed genes in three modules from the *Stra8*-deficient group. (e) *Sub1* mRNA levels in the control and *Sub1* siRNA groups. (f) *Stk31* mRNA levels in the control and *Stk31* siRNA groups. (g) The percentage of oocytes in cysts and follicles in control and *Sub1* siRNA groups. (h) The percentage of oocytes in cysts and follicles in control and *Stk31* siRNA groups. (i) Changes of *Lhx8*, *Nobox*, and *Sohlh1* mRNA levels in control and *Sub1* siRNA groups. (j) Changes of *Lhx8*, *Nobox*, and *Sohlh1* mRNA levels in control and *Stk31* siRNA groups. The percentage of each group is presented as the mean ± SD. All experiments were repeated at least three times (*$p < 0.05$; **$p < 0.01$, ***$p < 0.001$).
Supplementary Figure S5. *Mdk*-siRNA experiment verification.
(a) *Mdk* mRNA levels in the control and *Mdk* siRNA groups. (b) *Sdc1* mRNA levels in the control and *Sdc1* siRNA groups. (c) The number of oocytes per section in control and *Mdk* siRNA groups. The percentage of each group is presented as the mean ± SD. All experiments were repeated at least three times (*$p < 0.05$; **$p < 0.01$, ***$p < 0.001$).
Supplementary Table S1. Overview of the mapping parameters for the 10× Genomics scRNA-seq datasets established for mice ovaries.
