# Supplementary figures and images for: Single-Cell Transcriptomic Analysis of the Potential Mechanisms of Follicular Development in Stra8-Deficient Mice
Source: Int J Mol Sci. 2025 Apr 15;26(8):3734. doi: 10.3390/ijms26083734 (PMC12027774; doi:10.3390/ijms26083734)

Supplementary Figure S1

a

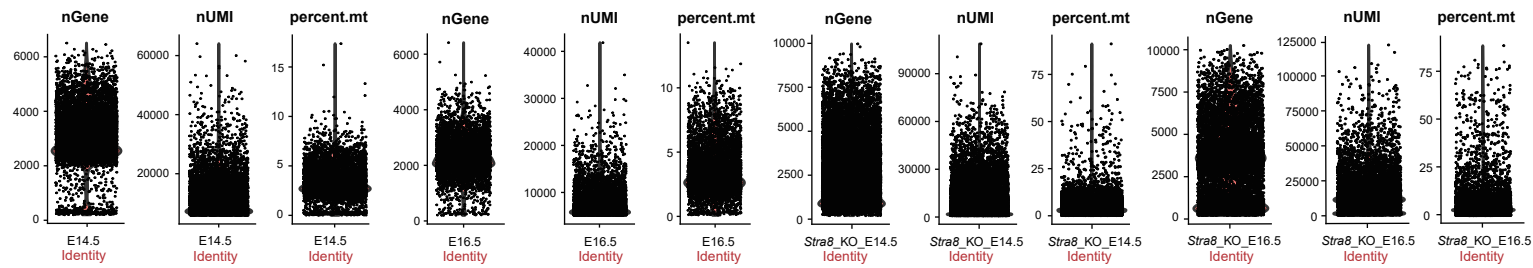

b

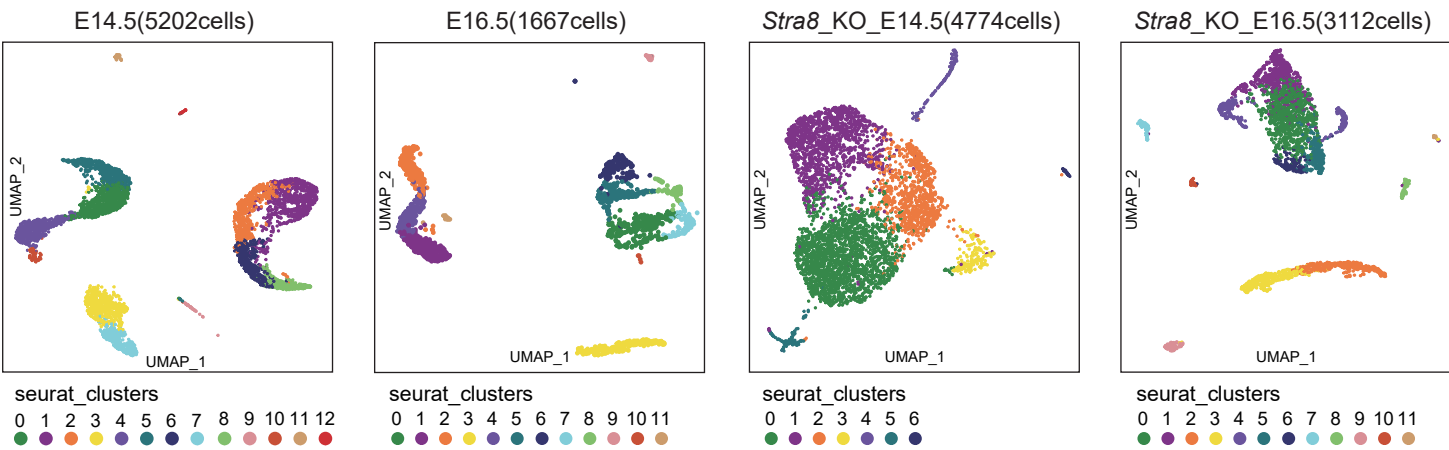

c

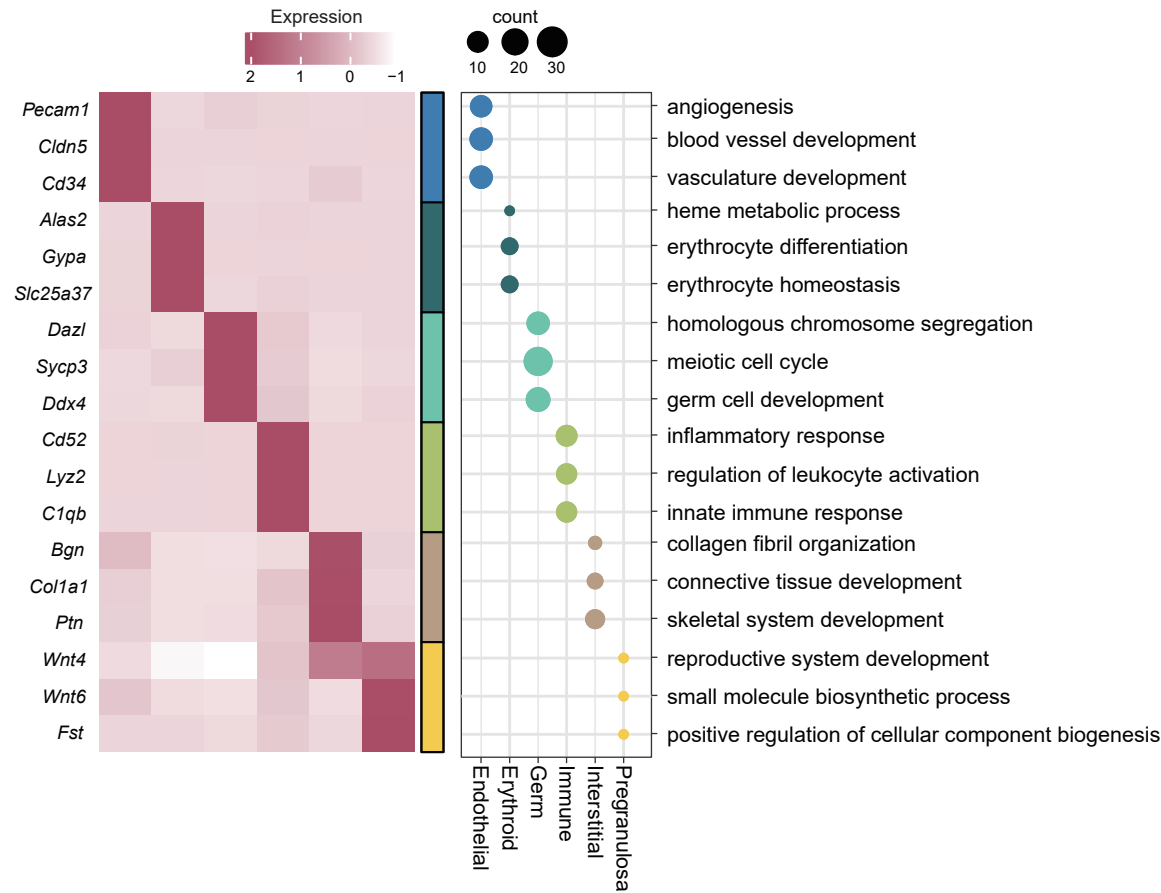

Supplement: Supplementary file 1 [file ijms-26-03734-s001.zip › Figure S1.pdf]

# Supplementary Figure S2

a

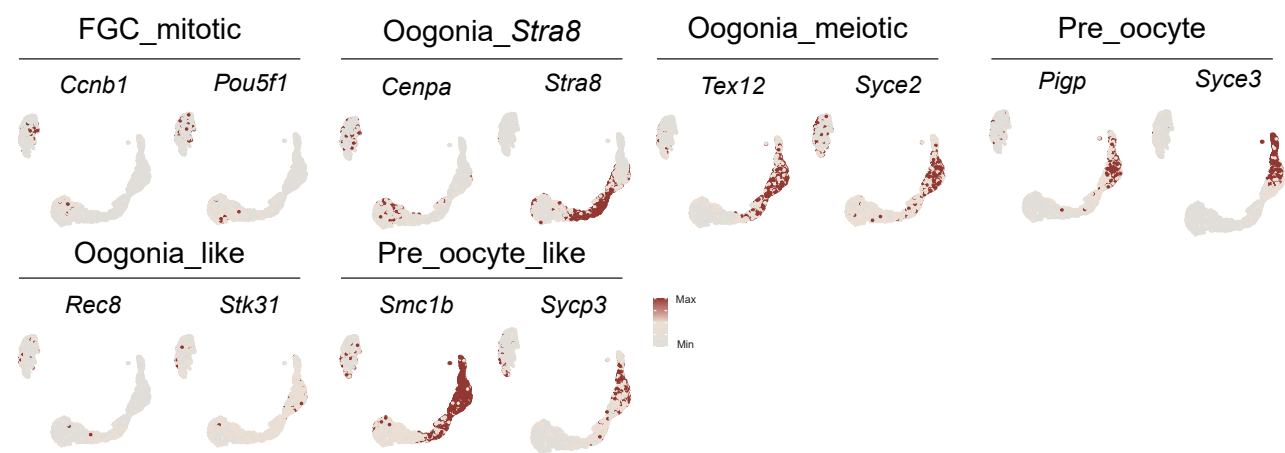

b

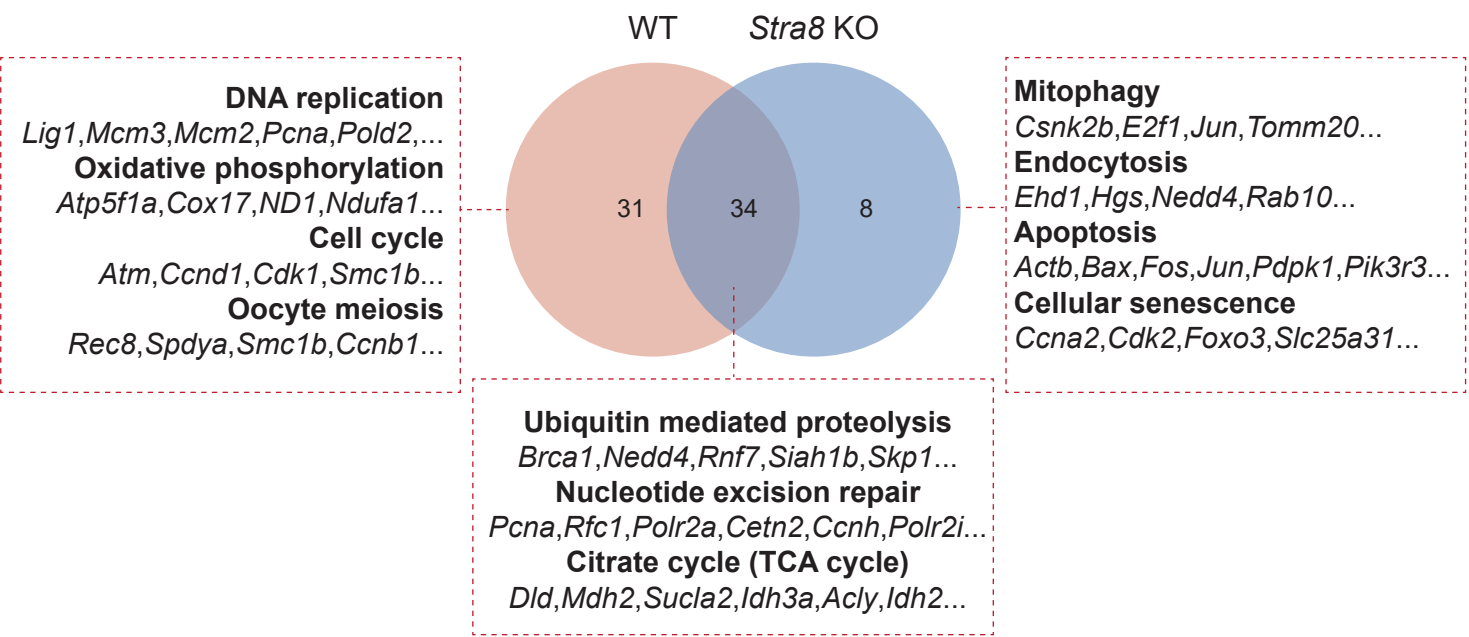

Supplement: Supplementary file 1 [file ijms-26-03734-s001.zip › Figure S2.pdf]

# Supplementary Figure S3

a

WT germ cell gene expression

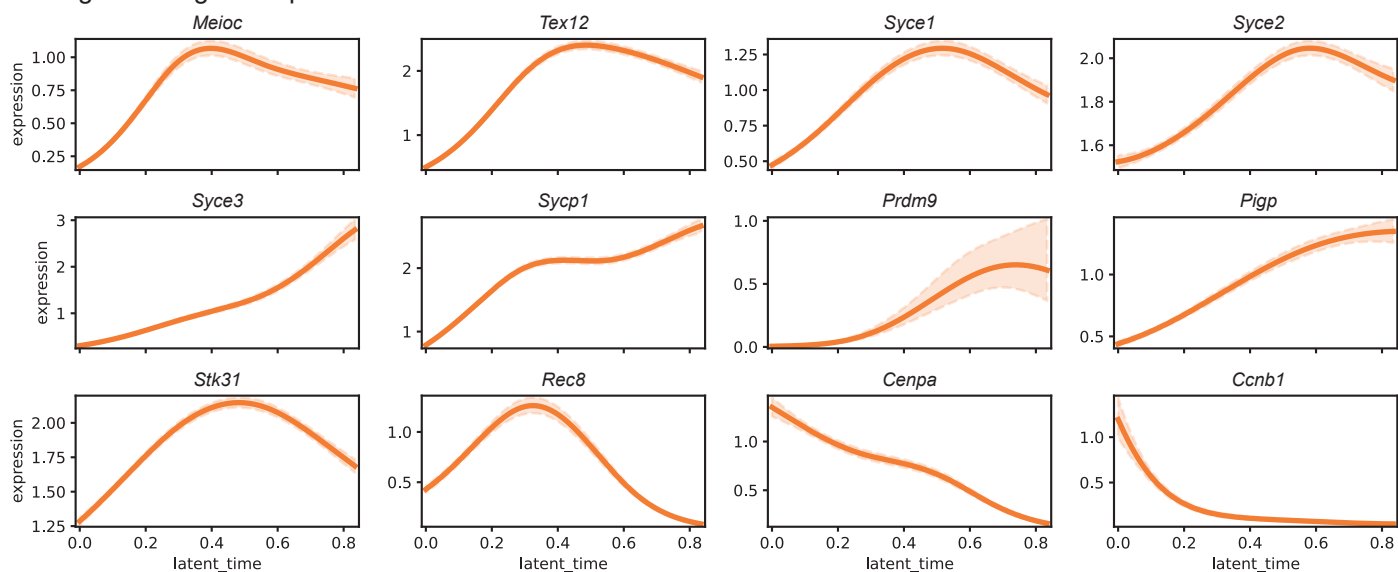

b

*Stra8* KO germ cell gene expression

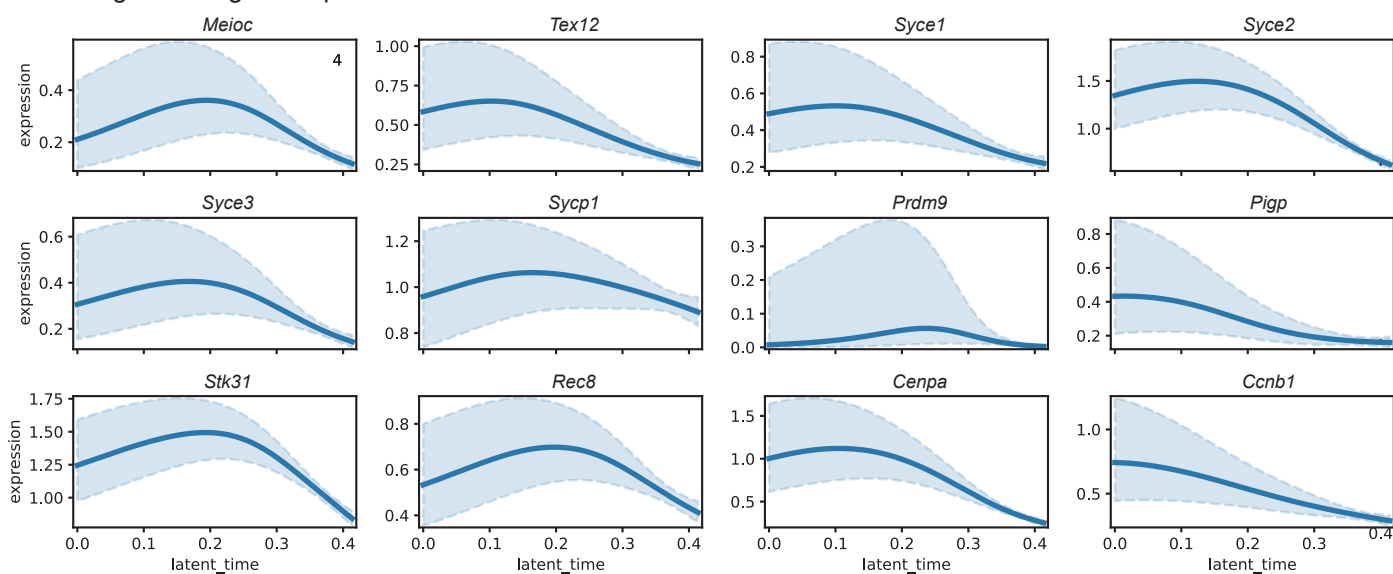

Supplement: Supplementary file 1 [file ijms-26-03734-s001.zip › Figure S3.pdf]

Supplementary Figure S4

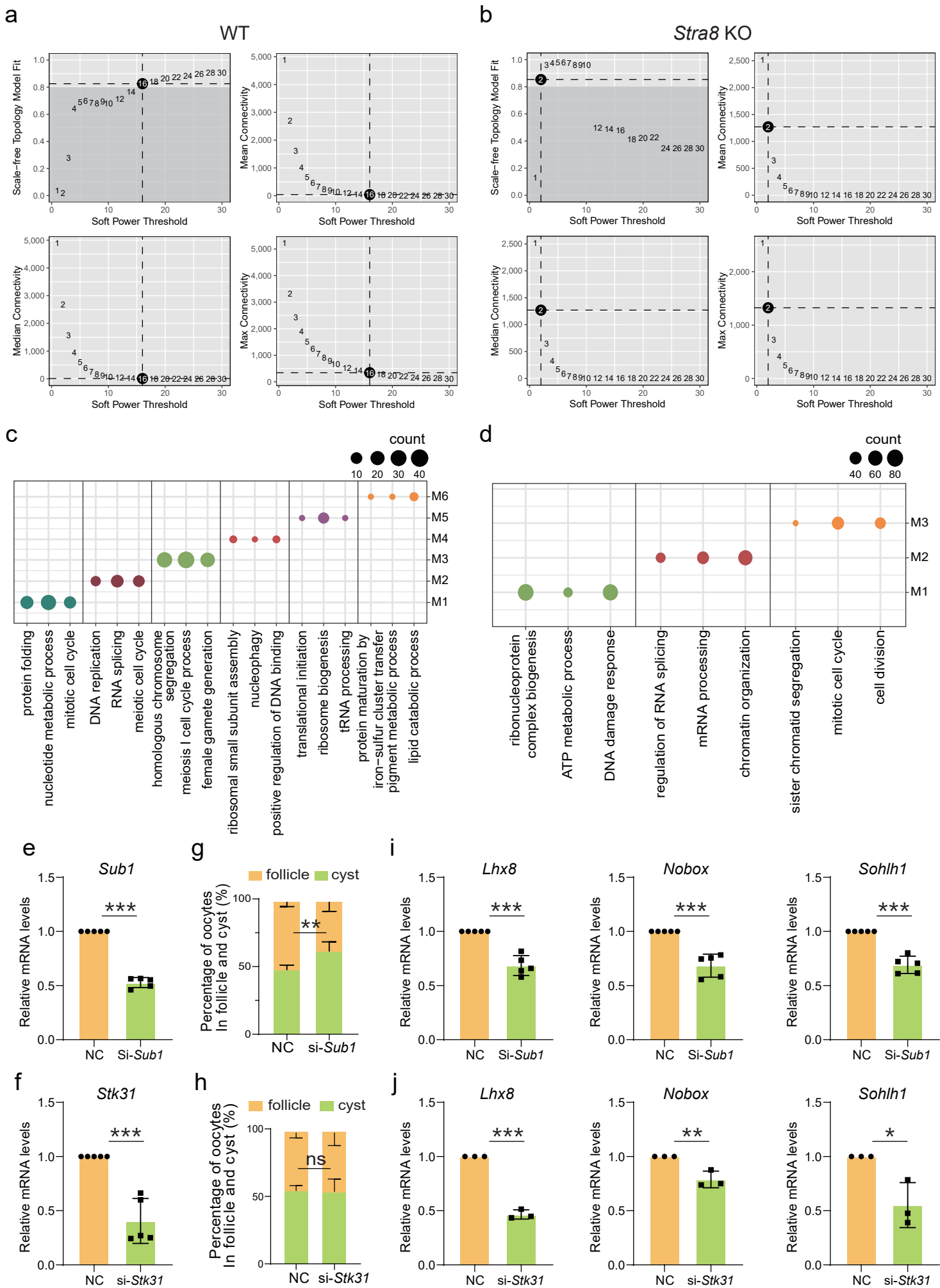

Supplement: Supplementary file 1 [file ijms-26-03734-s001.zip › Figure S4.pdf]

# Supplementary Figure S5

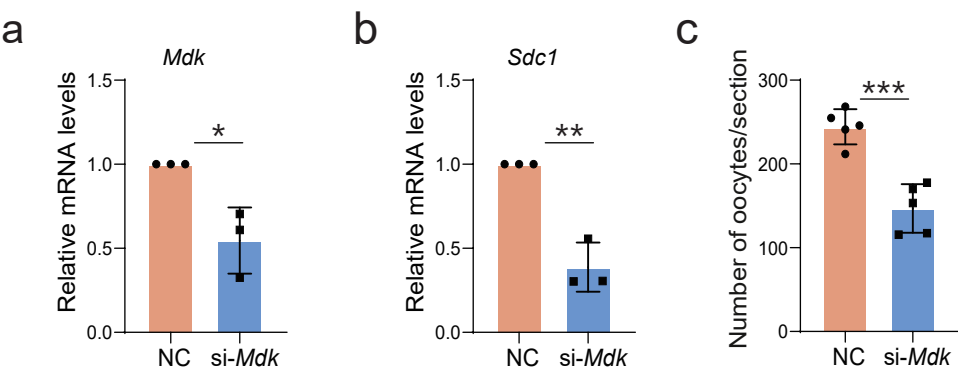

Supplement: Supplementary file 1 [file ijms-26-03734-s001.zip › Figure S5.pdf]
